# pH/redox sensitive nanoparticles with platinum(iv) prodrugs and doxorubicin enhance chemotherapy in ovarian cancer†

**DOI:** 10.1039/c9ra04034j

**Published:** 2019-07-02

**Authors:** Guyu Zhang, Yimin Zhu, Yushu Wang, Dengshuai Wei, Yixin Wu, Liuchun Zheng, Huimin Bai, Haihua Xiao, Zhenyu Zhang

**Affiliations:** Beijing Chaoyang Hospital Affiliated to Capital Medical University, Department of Gynaecological and Obstetric Beijing 100000 P. R. China zhenyuzhang2000@163.com; Affiliated Guangdong Medical University, Department of Oncology Zhanjiang 524000 P. R. China; Key Laboratory of Bio-based Material Science and Technology Ministry of Education, Northeast Forestry University Harbin 150040 P. R. China; Beijing National Laboratory for Molecular Sciences, State Key Laboratory of Polymer Physics and Chemistry, Institute of Chemistry, Chinese Academy of Sciences Beijing 100190 P. R. China hhxiao@iccas.ac.cn; University of Chinese Academy of Sciences Beijing 100049 P. R. China; College of Life Science and Technology, Beijing University of Chemical Technology Beijing 100029 P. R. China

## Abstract

pH/redox sensitive, dual drug loaded nanoparticles were prepared from poly(ethylene glycol)-*block*-poly(l-lysine) (PEG-*b*-PLL) for improving cancer therapy. Platinum(iv) and *cis*-aconitic anhydride-doxorubicin (CAD) were anchored to lysine residual amine groups of PLL to form polymer prodrug conjugates, which then self-assembled into nanoparticles with hydrophobic platinum(iv) prodrugs and CAD as the core. The nanoparticles were stable in neutral environments, but once under acidic and reductive conditions, the drugs were rapidly released. The dual-loaded nanoparticles had comparable intracellular toxicity to the regimen of combined application of free cisplatin and doxorubicin.

## Abstract 2

pH/redox sensitive, dual drug loaded nanoparticles were prepared from poly(ethylene glycol)-*block*-poly(l-lysine) (PEG-*b*-PLL) for improving cancer therapy.

## Graphical abstract

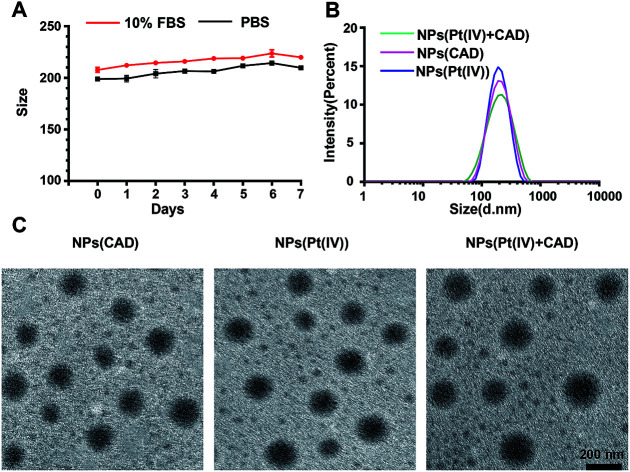

## 1 Introduction

**Scheme 1:**
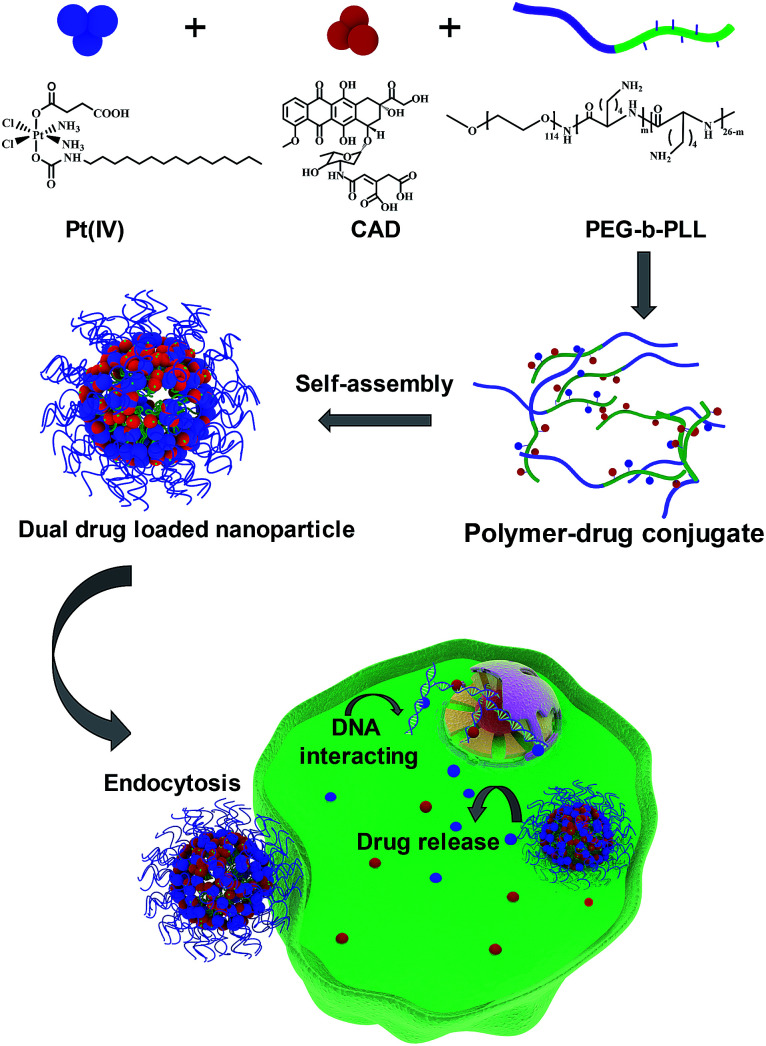
Preparation process of NPs (Pt(iv) + CAD) and the cell killing mechanism.

Ovarian cancer is one of the most lethal gynaecological cancers with 22 530 new cases and 13 980 associated mortalities reported in 2018.^1^ Combination chemotherapy of cisplatin (Pt) and doxorubicin (Dox) has been widely used as an anti-cancer regimen.^2,3^ In clinical trials, Pt and Dox were combined for recurrent ovarian cancer.^4^ However, the high toxicity of Dox and drug resistance against Pt caused by glutathione (GSH) compromised the therapy outcomes.^5–7^ One possible way to overcome these obstacles is to modify prodrugs with specific linkages cleaved under the intracellular environment of tumors, such as low pH^8^ and abundant glutathione,^9^ inducing cytoplasmic drug release. A Pt(iv) prodrug was considered as a promising agent to overcome platinum resistance. Xiao *et al.* reported that inert Pt(iv) prodrugs could transform into toxic Pt(ii) through abundant intracellular GSH and then kill the cancer cells.^10,11^ Herein, we developed a Pt(iv) prodrug with a long lipid chain (C_16_) and carboxyl group in the axial positions (Scheme S1†). CAD has also been proved to exhibit pH sensitive features^12^ (Scheme S2†). Poly(ethylene glycol)-*block*-poly(l-lysine) (PEG-*b*-PLL) has been widely used as polymer drug carrier due to its superior biocompatibility and stability.^13,14^ Hence, both Pt(iv) and CAD could be coupled with the amine groups of PLL block *via* amidation to self-assemble into nanoparticle (NPs (Pt(iv) + CAD)) (Scheme S3†). Prodrug nanoparticles emerged as promising candidates to deliver multiple anti-cancer agents.^15–17^ Compared with conventional chemotherapy, these nanometer-sized drugs exhibited considerable advantages, such as passive enhanced permeability and retention (EPR) impact on tumors, improved stability and solubility as well as lower toxicity in the bloodstream.^18–20^ When the pH/redox dual sensitive nanoparticles were internalized by tumor cells, Pt(iv) and CAD could be rapidly released under the acidic environment of tumor cells, and then turned into Pt(ii) and Dox to kill tumor cells. Robust cell-killing effect on ovarian cancer cells could be achieved with NPs (Pt(iv) + CAD) through synergistic effects (Scheme 1). Additionally, the pH/redox sensitive bonds of Pt(iv) prodrugs and CAD limited non-specific drug release in normal cells and alleviated the non-specific cell killing.

## 2 Results

### 2.1. Synthesis and characterization of pH/redox sensitive co-delivery nanoparticles

**Fig. 1:**
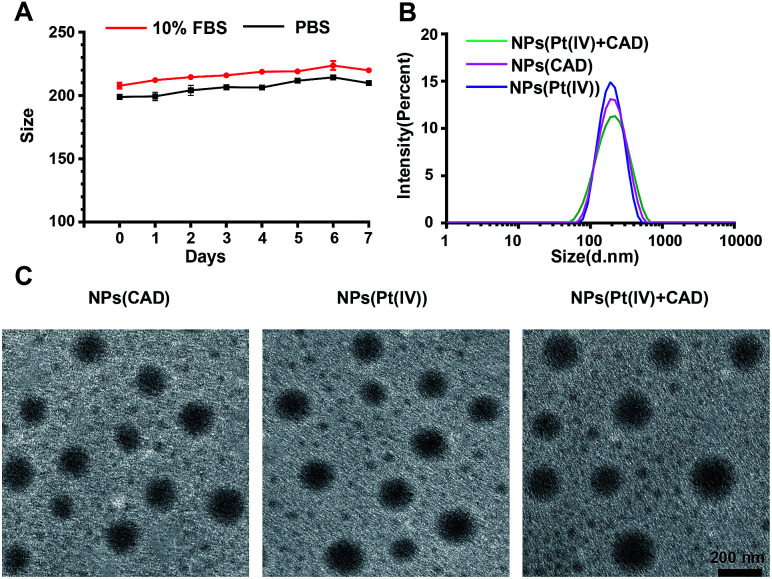
Characterization of nanoparticles. Stability of NPs (Pt(iv) + CAD) (A). DLS of nanoparticles (B) and TEM images of nanoparticles (C). Scale bar: 200 nm.

In this study, we prepared a biocompatible pH/redox sensitive nanoparticle for co-delivery of CAD and Pt(iv) prodrugs. The CAD or Pt(iv) were conjugated with the amine groups of PLL *via* 1-(3-dimethylaminopropyl)-3-ethyl carbodiimide hydrochloride (EDC) and *N*-hydroxysuccinimide; 1-hydroxypyrrolidine-2,5-dione (NHS) mediated amidation reaction (Schemes S4 and S5†). The maximum Pt loading (8%) was achieved when drug to polymer molar reached 8 : 1, the optimal Dox loading was 10% when drug to polymer molar ratio was at 6 : 1 (Fig. S1†). After a series of optimizations, drugs to polymer molar ratio (Pt : Dox : PEG-*b*-PLL) of 8 : 6 : 1 was selected as the optimized ratio to synthesize the NPs (Pt(iv) + CAD) in the following study. Eventually, the NPs (Pt(iv) + CAD) with Pt to Dox ratio at 2 : 1 were obtained. All the synthesized prodrugs and copolymers were confirmed by using ^1^H-NMR (Fig. S4–S6†). Furthermore, the NPs (Pt(iv) + CAD) showed great size stability under PBS and 10% fetal bovine serum (Fig. 1). The size and PDI of nanoparticles are summarized in Table 1, suggesting the nanoparticles have been prepared.

**Table 1:** The size and PDI of NPs (Pt(iv) + CAD), NPs (Pt(iv)) and NPs (CAD)

	Particle size (nm)	PDI
NPs (Pt(iv) + CAD)	198.6 ± 14.0	0.21
NPs (Pt(iv))	194.5 ± 10.1	0.2
NPs (CAD)	185.7 ± 7.1	0.18

### 2.2. Cellular uptake of platinum and Dox and intracellular drug localization

**Fig. 2:**
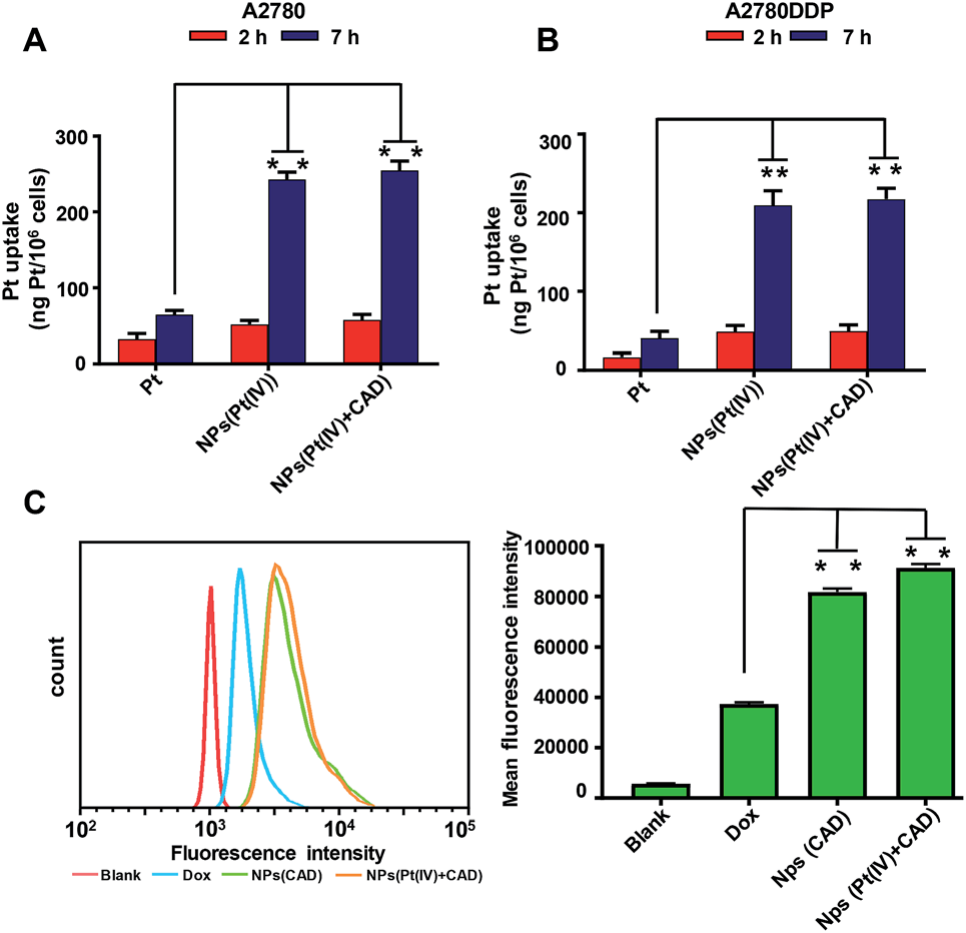
*In vitro* cellular uptake of platinum and doxorubicin. Quantification of the platinum uptake on A2780 (A), A2780DDP (B) by ICP-MS. Quantification of the Dox uptake on A2780DDP by flow cytometry (C). Significance is defined as **P < 0.001, *P < 0.01.

**Fig. 3:**
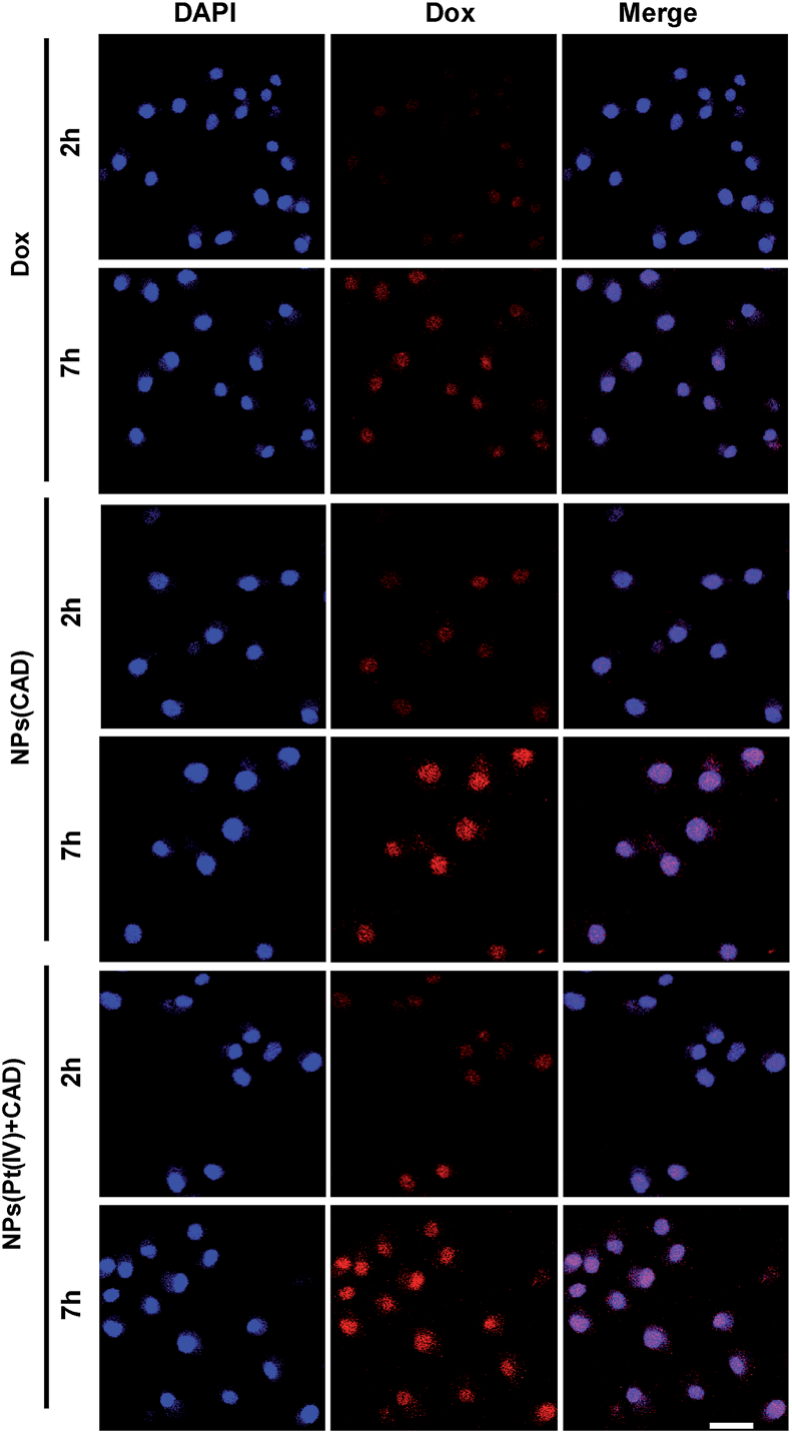
CLSM images of A2780DDP incubated with free Dox and nanoparticles for 2 h and 7 h. The blue fluorescence comes from a nuclear dye DAPI. The red fluorescence comes from Dox. Scale bar: 20 μm.

The uptake of platinum by A2780 and A2780DDP (both cell lines were obtained from the Medical Department of Jilin University in China) was qualified by ICP-MS after incubation with cells for 2 h and 7 h. As shown in Fig. 2, the process of cell uptake was time dependent. The result shows that the amount of platinum endocytosis for NPs (Pt(iv) + CAD) and NPs (Pt(iv)) at 7 h were 3.5 to 4 times more than free Pt on A2780 and A2780DDP. No obvious difference was observed between NPs (Pt(iv) + CAD) and NPs (Pt(iv)). Flow cytometry was here applied to monitor the internalization of Dox after 7 h treatment. Compared with free Dox, the fluorescence of nanoparticles group shifted right significantly. Up to three times enhancement of intercellular uptake was achieved on nanoparticles group. To further visualize the endocytosis pathway of nanoparticles, confocal laser scanning microscopy (CLSM) was applied to locate the nanoparticles. In Fig. 3, after incubation with free Dox and nanoparticles for 2 h and 7 h respectively, red signal was mainly presented in the nucleus of A2780DDP. The uptake pattern of nanoparticles was in a time dependent manner as the red fluorescence intensity of 7 h incubation was brighter than 2 h in all groups, which was consistent with the result of uptake *via* ICP-MS shown in Fig. 2. Meanwhile, the red fluorescence signal of nanoparticles group was stronger than free Dox, suggesting the nanoparticles could facilitate the internalization process of prodrug.

### 2.3. pH/redox responsive drug release

**Fig. 4:**
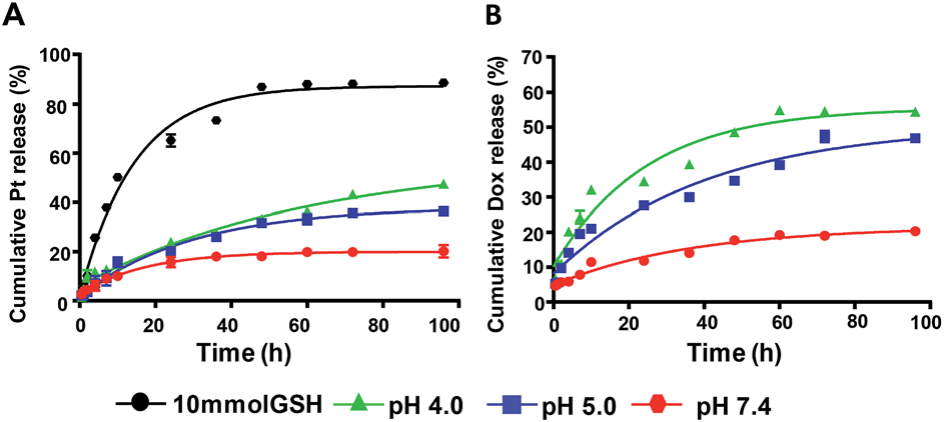
The drug release profile of NPs (Pt(iv) + CAD). The platinum release under different conditions (A), the Dox release under different conditions (B).

To investigated the triggered release property of nanoparticles, cumulative platinum and Dox release *in vitro* from nanoparticles were observed at pH 7.4, pH 5.0, pH 4.0 and in 10 mmol GSH solutions, respectively. At pH 7.4, which was similar to pH value of normal tissue and circulatory system, the platinum release is less than 20% up to 40 h, while in the reductive environment such as in GSH solution, platinum release rate accelerated significantly and reached almost 90% after incubation for 48 h. Correspondingly, the Dox release was almost retarded under neutral environment, the cumulative release rate was 20% after incubation for 96 h. However, at pH 5.0 and 4.0, the release rate of Dox was elevated as the pH decreased. The cumulative Dox release rate ultimately increased to 40% and 50%, respectively. This result indicated that acidic and reductive environment enhanced the cleavage of the amide bond between CAD prodrugs and PLL and induced Dox and Pt release (Fig. 4).

### 2.4. *In vitro* cytotoxicity

**Fig. 5:**
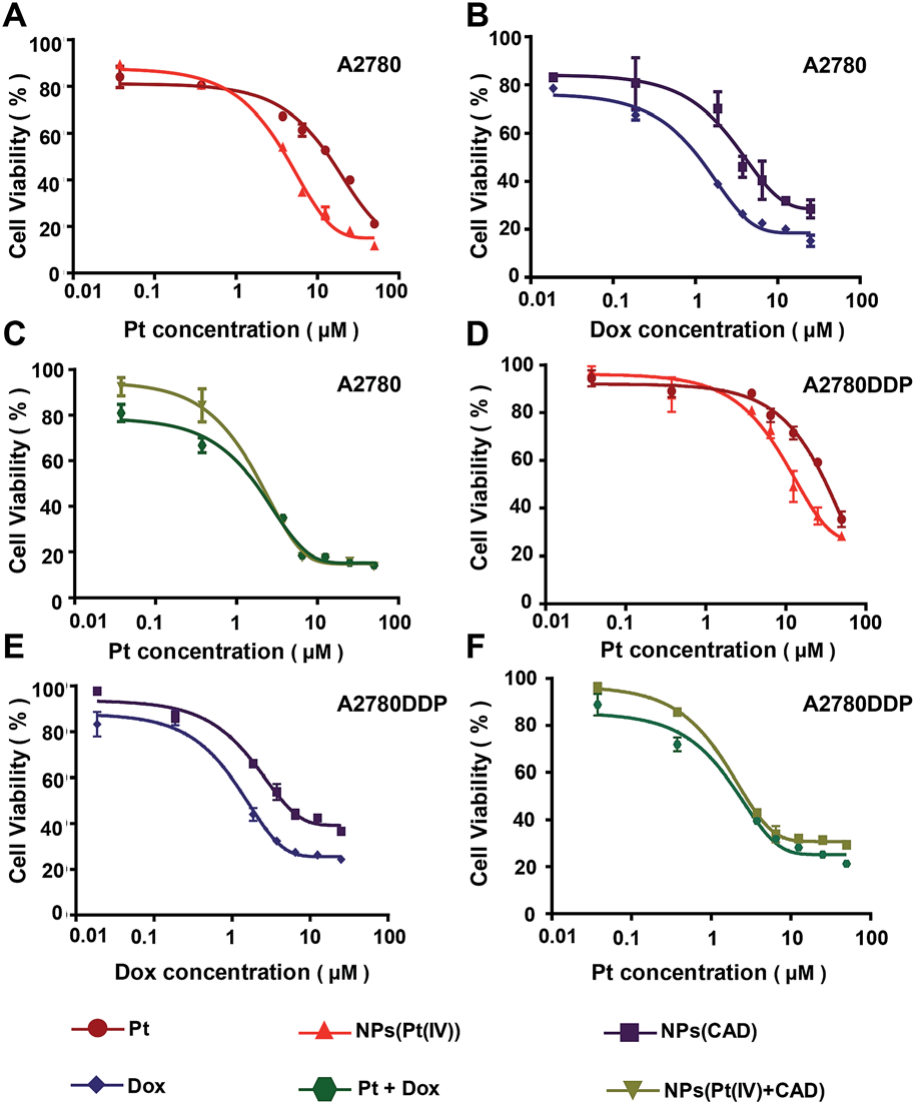
*In vitro* evaluation of anticancer activity of nanoparticles. The cytotoxicity of Pt *vs.* NPs (Pt(iv)) (A) and (D), NPS (CAD) *vs.* Dox (B) and (E), Pt + Dox *vs.* NPs (Pt(iv) + CAD) (C) and (F) to A2780 and A2780DDP.

The cytotoxicity of Pt, Dox, Pt + Dox, NPs (Pt(iv)), NPs (CAD) and NPs (Pt(iv) + CAD) were evaluated by MTT assay on A2780 cells and Pt resistant cells A2780DDP. In Fig. 5, NPs (Pt(iv)) significantly increased cytotoxicity (IC_50_ = 3.80 μM, IC_50_ = 9.61 μM) compared to free Pt (IC_50_ = 9.41 μM, IC_50_ = 31.03 μM) to A2780 and A2780DDP. However, the NPs (CAD) (IC_50_ = 3.84 μM, IC_50_ = 5.7 μM) manifested decreased drug potency compared to free Dox (IC_50_ = 1.08 μM, IC_50_ = 1.10 μM), suggesting NPs (CAD) could attenuate the toxicity. For the combination of free Pt and Dox (IC_50_ = 0.75 μM, IC_50_ = 0.61 μM) and the NPs (Pt(iv) + CAD) (IC_50_ = 0.72 μM, IC_50_ = 0.60 μM), they almost exhibited similar toxicity against ovarian cancer cells. The Combination Index (CI) was applied here to assess the synergistic effect of the NPs (Pt(iv) + CAD) as well as Pt + Dox, the CI values of higher than, equal to, and lower than 1 represented antagonism, additivity, and synergism, respectively.^21^ The IC_50_ value of each group and CI of NPs (Pt(iv) + CAD) as well as Pt + Dox were summarized in Table 2, showing robust synergy effects for NPs (Pt(iv) + CAD) that were achieved in A2780 and A2780DDP cell line (CI_50_ = 0.55, CI_50_ = 0.21).

**Table 2:** The summary of IC_50_ and CI index. ★ represents IC_50_ based on Pt. ◆ represents IC_50_ based on Dox. N.A. represents not applicable

	IC_50_		CI_50_	
	A2780	A2780DDP	A2780	A2780DDP
Pt	9.41	31.03	N.A.	N.A.
Dox	1.08	1.10	N.A.	N.A.
Pt + Dox	1.22^★^/0.75^◆^	0.99^★^/0.61^◆^	0.87	0.59
NPs (Pt(iv) + CAD)	1.38^★^/0.72^◆^	0.99^★^/0.60^◆^	0.55	0.21
NPs (Pt(iv))	3.80	9.61	N.A.	N.A.
NPs (CAD)	3.84	5.7	N.A.	N.A.

### 2.5. Cell cycle and apoptosis analysis

**Fig. 6:**
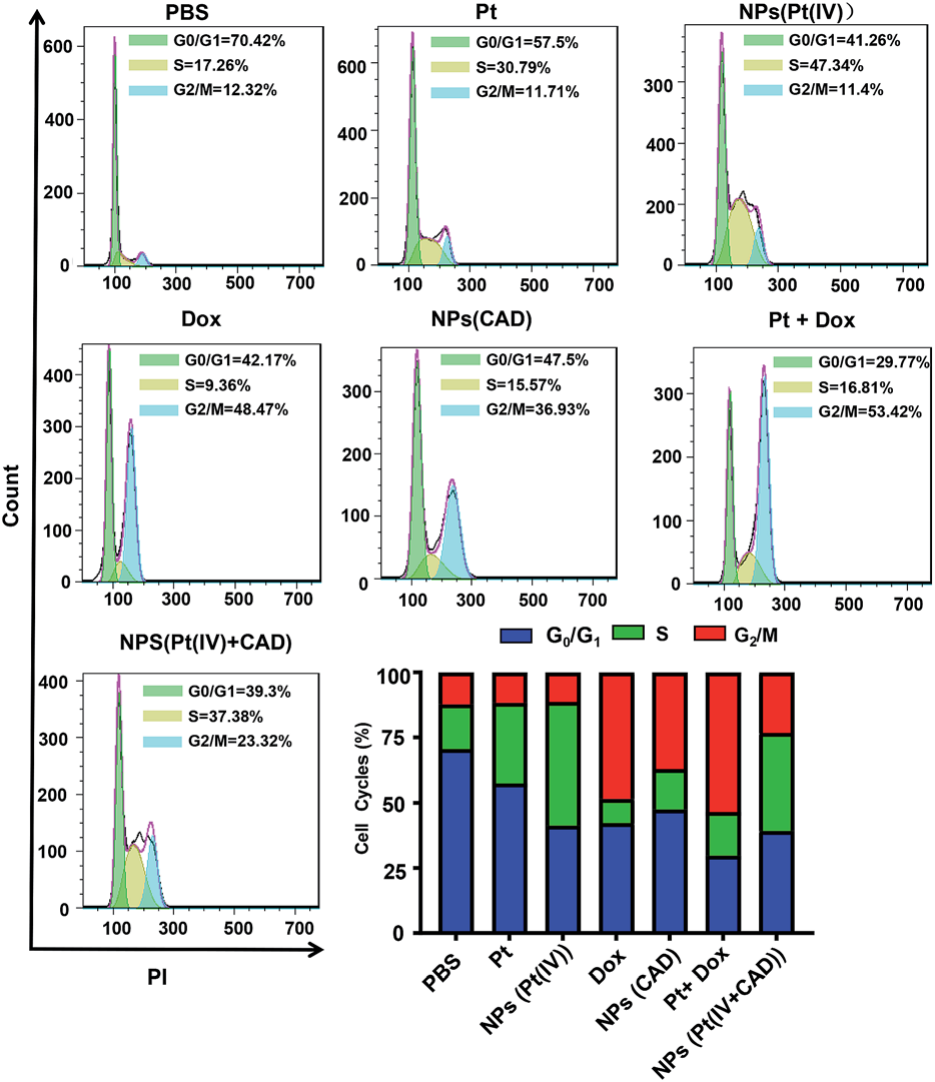
Cell cycles of free Pt, Dox, and, nanoparticles in A2780DDP. The cell cycle arrest induced by PBS, NPs (Pt(iv)), Pt, Dox, NPs (CAD), Pt + Dox, NPs (Pt(iv) + CAD).

**Fig. 7:**
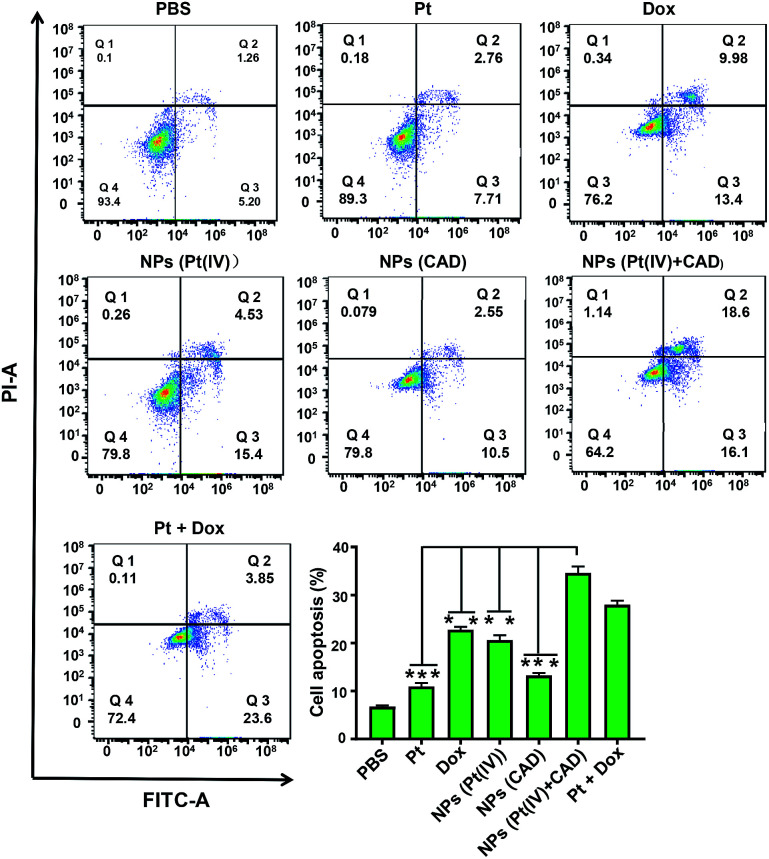
Cell apoptosis of Pt, Dox, and nanoparticles in A2780DDP. The cell apoptosis induced by NPs (Pt(iv) + CAD), Pt + Dox, NPs (Pt(iv)), Cis, NPs (CAD), Dox and PBS. Significance is defined as **P < 0.001, *P < 0.01.

To clarify the mechanism of cell death induced by NPs (Pt(iv) + CAD), the cell cycle and apoptosis analysis were carried out *via* flow cytometry. During the normal cell cycle, G_1_ phase initiated growth-dependent cyclin-dependent kinase (CDK) activity and promoted the transition of G_1_ to S phase. S phase mainly contributed to the duplication of DNA, and most of the RNA and protein was synthesized during the G_2_/M phase.^22^ As presented in Fig. 6, the free Pt and free Dox led to increased cell cycle arrest in the S phase and G_2_/M phase respectively, which was consistent to previous studies.^23,24^ The S phase arrest of NPs (Pt(iv)) was superior to free Pt treatment. On the other hand, the G_2_/M phase arrest of free Dox was stronger than NPs (CAD). As compared to PBS group, we observed that the ratio of cells in both S phase and G phase increased significantly for NPs (Pt(iv) + CAD) groups, indicating that they could induce cell apoptosis by interfering with the synthesis of DNA. Correspondingly, in Fig. 7, the cell apoptotic study for A2780DDP was also carried out. The result suggested that the NPs (Pt(iv) + CAD) were more effective in promoting cell apoptosis than single free drug or single-drug loaded nanoparticles.

## 3 Conclusion

pH/redox-sensitive nanoparticles with dual loaded prodrugs, NPs (Pt(iv) + CAD), were successfully prepared in this study. The hydrophobic segment of Pt(iv) prodrugs and CAD were not only the core of NPs (Pt(iv) + CAD), but also remained stable, which resulted in low drug release of both drugs under the neutral condition. However, the NPs (Pt(iv) + CAD) could efficiently release platinum and Dox under acidic environment with high GSH concentration, which was the main characteristics for tumour cells. Furthermore, great synergistic effect was achieved on the NPs (Pt(iv) + CAD). Ovarian cancer cells treated with NPs (Pt(iv) + CAD) demonstrated increased S and G_2_/M phase arrest for cell cycles, resulting in the apoptosis of cancer cells. Therefore, a promising and efficient anti-cancer therapy has been achieved with NPs (Pt(iv) + CAD).

## Conflicts of interest

There are no conflicts to declare.

## Supplementary Material

RA-009-C9RA04034J-s001
