# Supplementary material for: pH/redox sensitive nanoparticles with platinum(iv) prodrugs and doxorubicin enhance chemotherapy in ovarian cancer
Source: RSC Adv. 2019 Jul 2;9(36):20513–7. doi: 10.1039/c9ra04034j (PMC9065746; doi:10.1039/c9ra04034j)
Supplement: RA-009-C9RA04034J-s001 [file RA-009-C9RA04034J-s001.pdf]

Electronic Supplementary Material (ESI) for RSC Advances.
This journal is © The Royal Society of Chemistry 2019

Electronic Supplementary Information (ESI)

pH/redox sensitive nanoparticles with Platinum (IV) prodrugs and doxorubicin enhance chemotherapy in ovarian cancer

Guyu Zhang, [a] Yimin Zhu, [b] Yushu Wang, [c] Dengshuai Wei, [d, e] Yixin Wu , [f] Liuchun Zheng, [e] Huimin Bai, [a, *] Haihua Xiao, [d, e, *] Zhenyu Zhang, [a, *]

a. Beijing chaoyang hospital affiliated to capital medical university, department of gynaecological and obstetric, Beijing 100000, P.R. china
b. Affiliated Guangdong medical university, department of oncology, Zhanjiang 524000, P.R. china
c. Key Laboratory of Bio-based Material Science and Technology Ministry of Education, Northeast Forestry University, Harbin 150040, P.R. China.
d. Beijing National Laboratory for Molecular Sciences, State Key Laboratory of Polymer Physics and Chemistry, Institute of Chemistry, Chinese Academy of Sciences, Beijing 100190, P.R. China
e. University of Chinese Academy of Sciences, Beijing 100049, P.R. China.
f. College of life Science and Technology, Beijing university of Chemical Technology Beijing 100029, P.R. china

TABLE OF CONTENTS

Experimental ………………………………………..……………………………………………..……4-10

Scheme ……………………………………………………………………………………………......11-13

Figure S1……………………………………………………………………………………………...13

Figure S2………………………………………………………………………………………………14

Figure S3………………………………………………………………………………………………15

Figure S4………………………………………………………………………………………………16

Figure S5………………………………………………………………………………………………17

Figure S6……………………………………………………………………………………….…...18

Figure S7………………………………………………………………………………………………19

Figure S8………………………………………………………………………………………………20

Experimental material and instrument

Materials

Methoxyl-poly (ethylene glycol) - block - poly (polylysine) ($mPEG_{114}$-b-PLL_{26}) (MW≈8500) was gifted by Changchun Institute of Applied Chemistry. Cisplatin were purchased from Shangdong Boyuan Pharmaceutical *Co Ltd.*, doxorubicin hydrochloride was purchased from aladdin. Cis-aconitic anhydride, hexadecyl isocyanate and 2-(4-amidinophenyl)-1H-indole-6-carboxamidine (DAPI) and dodecyl isocyanate were purchased from Sigma. RPMI 1640, fetal bovine serum (FBS), penicillin-streptomycin solution, trypsin, Annexin V-FITC/PI apoptosis detection kit, and cell cycle detection kit were purchased from Keygen Biotech. Hydrogen peroxide, N, N-Dimethylformamide (DMF) were purchased from Beijing chemical works.

Instruments

Dynamic light scattering (DLS) was conducted by Malvern Zetasizer NanoZS90. Transmission electron microscopy (TEM) was performed by using JEM-1011 electron microscope operated at 100 kV. All OD values were measured by Spectra Max M3. Flow cytometry was administered by Cytomics FC500 Flow Cytometry (Beckman Coulter Ltd.). Confocal laser scanning microscopy (CLSM) were performed with ZEISS LSM880. ^1H NMR spectra was recorded in DMSO-d_6 on a 400 MHz NMR spectrometer (Bruker) at 298 K. Inductively coupled plasma mass spectrometry (ICP-MS) was performed by Agilent technologies 7700 series. Matrix-assisted laser desorption/ionization time-of-flight mass spectrometry (MALDI-TOFMS) were obtained by using an Autoflex III (Bruker). High resolution mass spectrometry (HR-

MS) was conducted by Agilent 1290 UPLC/6540 Q-TOF

Preparation of Pt(IV)-OH

Cisplatin (0.5 g, 1.67 mmol) was dissolved in H_2O_2 (30% w/v, 20 mL). The mixture was stirred at 50 ℃ overnight and clear solution was produced. After the mixture drop down to the room temperature, a large number of needle-like crystal was precipitated. The product was washed several times with acetone or H_2O in turn and dried in vacuum oven. The yield rate was 80%.

Preparation of Pt (IV)-COOH

Pt(IV)-OH (0.3 g, 0.9 mmol) was suspended in Dimethylformamide (DMF) (20 mL), and then succinic anhydride (0.09 g, 0.9 mmol) was added. The mixture was under reaction at room temperature for 24 h. The solution was evaporated and then 10 mL diethyl ether was added to precipitate a light yellow solid. Finally, the precipitation was washed several times with acetone, diethyl ether, and then dried.

Preparation of C_{16}-Pt (IV)-COOH

Hexadecyl isocyanate (160.47 mg, 0.6 mmol) was added to Pt (IV)-COOH (260.478 mg, 0.6 mmol) and suspended in 10 mL DMF. The solvent was removed under reduced pressure after reaction at 65 °C for 12 h, and then the residue was recrystallized by MeOH. The final product is a yellow solid and confirmed by ^1H NMR (Figure S2). 1H NMR (300 MHz, DMSO) δ 6.58 (d, J = 32.2 Hz, 7H), 2.88 (d, J = 6.4 Hz, 2H), 2.46 – 2.34 (m, 2H), 2.33 – 2.17 (m, 2H), 1.28 (d, J = 29.0 Hz, 28H), 0.85 (t, J = 6.0 Hz, 3H).

Preparation of cis-aconitic anhydride doxorubicin (CAD)

Doxorubicin Hydrochloride (0.464 g, 0.8 mmol) and Cis-Aconitic (0.1372 g, 0.88 mmol) was suspended in in 20 mL of anhydrous DMF, and then 134 μl of triethylamine was added. The mixture was stirred overnight at room temperature in the dark environment under nitrogen (N_2) atmosphere. Next, 200 mL cold ethyl acetate was added to the mixture, and washed first with a saturated sodium chloride solution at pH 2-3 and then with a saturated solution at pH 7.4. The obtained organic layer was dehydrated with anhydrous sodium sulfate for 24 h. Finally, solid Cis-Aconitic anhydride was extracted by filtering and dried under vacuum at room temperature, the final product was a red powder (0.3g, 50%).^{1}H NMR (400 MHz, DMSO) δ 14.05 (s, 1H), 13.28 (s, 1H), 9.79 (s, 2H), 8.43 (s, 1H), 7.92 (s, 2H), 7.66 (s, 1H), 5.47 (s, 1H), 5.29 (d, J = 30.6 Hz, 1H), 5.00-4.82 (m, 2H), 4.59 (s, 2H), 4.19 (s, 1H), 4.03 (s, 1H), 4.00 (s, 3H), 3.55 (d, J = 30.0 Hz, 2H), 2.90 (s, 2H), 2.17 (d, J = 22.3 Hz, 2H), 1.90 (s, 2H), 1.14 (d, J = 8.4 Hz, 3H).

Synthesis of PEG_{114}-b-PLL_{26}-Pt (IV)-CAD and preparation of the nanoparticle

C_{16}-Pt (IV)-COOH (56 mg) and CAD (42 mg) were dissolved into DMSO (500 μl), respectively, and then 1-(3-Dimethylaminopropyl)-3-ethylcarbodiimide hydrochloride (EDC) (120 mg) and N-Hydroxysuccinimide;1-hydroxypyrrolidine-2,5-dione (NHS)(100 mg) were add to each mixture and stirred for 2 hours. After the above steps, two kind of mixture were mixed together, and 80 mg PEG_{114}-b-PLL_{26} were added and continuously reacted for two days. Finally, the mixture was collected

and dialyzed against a dialysis bag with molecular weight of 3,500 for 48 h, and the supernatant was collected by centrifugation (4000 rpm, 5 min).

Synthesis of PEG_{114}-b-PLL_{26}-Pt (IV) and preparation of the nanoparticle

C_{16}-Pt (IV)-COOH (56 mg) were dissolved into DMSO (500 μl), and then 1-(3-Dimethylaminopropyl)-3-ethylcarbodiimide hydrochloride (EDC) (120 mg) and N-Hydroxysuccinimide;1-hydroxypyrrolidine-2,5-dione (NHS) (100 mg) were add and stirred for 2 hours. After the above steps, 80 mg PEG_{114}-b-PLL_{26} were added and continuously reacted for four days. Finally, the mixture was collected and dialyzed against a dialysis bag with molecular weight of 3500 for 48 h, and the supernatant was collected by centrifugation (4000 rpm, 5 min).

Synthesis of PEG_{114}-b-PLL_{26}-CAD and Preparation of the nanoparticle

CAD (42 mg) were dissolved into DMF (500 μl), and then 1-(3-Dimethylaminopropyl)-3-ethylcarbodiimide hydrochloride (EDC) (120 mg) and N-Hydroxysuccinimide;1-hydroxypyrrolidine-2,5-dione (NHS) (100 mg) were add and stirred for 2 hours. After the above steps, 80 mg PEG_{114}-b-PLL_{26} were added and continuously reacted for four days. Finally, the mixture was collected and dialyzed against a dialysis bag with molecular weight of 3500 for 48 h, and the supernatant was collected by centrifugation (4000 rpm, 5 min).

Dynamic light scattering

The diameters of nanoparticles were performed by a Nano-ZS zeta sizer (Malvern Instrument Ltd., UK) with a laser light wavelength of 632.8 nm at a scattering angle at 173°. The zeta sizer was commonly calibrated with a 60 nm nanosphere™ standard (Duke Scientific Corp. CA). Each sample was operated three times, and then the DTS software version 3.32 were applied to process the results.

Drug release of NPs

Drug release of NPs was studied at pH 7.4, pH 5.0, pH 4.0 and in the presence of 10 mmol GSH, respectively. Solutions of different pH were prepared in PBS. Nanoparticles solution of 1 mL was taken into dialysis bag (MW=3,500) and then immersed in those solutions of different pH as soon as possible. Next, the systems were placed into an incubator shaking at 37 °C. At each specific point in time, 1 mL of sample solution was collected and replenish a considerable volume of solution immediately. All the samples were examined by ICP-MS and UV-vis spectrometry. The cumulative Pt and doxorubicin release were defined as the percentage of the cumulative Pt and doxorubicin in the dialysate to the platinum and doxorubicin in the nanoparticles.

Cell lines and cell incubation conditions

A2780 and A2780DDP were obtained from the Medical Department of Jilin University in China. A2780, A2780DDP cells were used in the following experiments. A2780, A2780DDP cells were cultured in RPMI 1640 media. Culture medium were supplemented with 10% (V/V) fetal bovine serum, 1% (V/V) 10 kU /mL penicillin

and 10 mg/mL streptomycin. The cell lines were cultured in 37 °C with % (V/V) CO_2 atmosphere.

Platinum uptake in the cells

A2780 and A2780DDP cells were seeded in six-well plates at a density of 1×10^6 per well and incubated at 37 °C for 12 h. Cisplatin and NPs with concentration of 10 μM/L of Pt were added into each well. After treatment for 2 h and 7 h, the cells were harvested in EP tubes and ICP-MS was performed to examine the Pt concentrations.

Doxorubicin uptake in the cells

A2780DDP cells were seeded in six-well plates at a density of 1×10^6 per well and incubated at 37 °C for 12 h. Free doxorubicin and NPs were added with concentration of 5 μM of doxorubicin were added into each well. After treatment for 2 h and 7 h, the cells were harvested in EP tubes and flow cytometry was performed to examine the doxorubicin concentrations.

Intracellular localization of the nanoparticles with confocal laser scanning microscopy (CLSM)

3×10^4 A2780DDP cells were incubated in cell culture dish designed for CLSM 37 °C and lasted for 12 h. Subsequently, the NPs (Pt (IV)+CAD), free doxorubicin, NPs (CAD) were added at a final concertation of 1 μM for 2 h,7 h. The nuclei were stained with DAPI. After washed with cold PBS, the cells were fixed with Paraformaldehyde. In the end, images were performed with CLSM.

Cell relative viability studies

MTT assay was used to examine the cell relative viability. A2780, A2780DDP

cells were seeded in 96-well plates with a density of 5×10^3 per well and cultured at 37 °C for 12 h. Cells were treated with cisplatin, doxorubicin, cisplatin + doxorubicin and NPs with final concentrations (μM) of 0.03125, 0.3125, 3.125, 6.25, 12.5, 25, 50 with Pt and 0.01562, 0.1562, 1.562, 3.125, 6.25, 12.5, 25 with doxorubicin respectively. Cells were treated with PEG_{114}-b-PLL_{26} with final concentrations (μg/ml) of 400,200,100,50,25,12.5. After treatment for 48 h, 10% MTT diluted with 1640 was added in the wells. After incubation in 37 °C for 4 h, 10% SDS was added in the wells and cells were incubated in 37 °C for 12 h in the dark. The results were performed with Molecular Devices.

Apoptosis studies

A2780 DDP cells were seeded in six-well plates at a density of 1×10^5 per well and incubated at 37 °C for 12 h. cisplatin, doxorubicin, cisplatin+ doxorubicin, NPs were added in the wells at the final concentration of 10 μM of Pt and 1 μM of doxorubicin. After treatment for 24 h, the media was removed and the cells were washed 3 times with cold PBS. Then the cells were harvested and strained with 5 μL FITC and 5 μL PI for 10 min in the dark at room temperature respectively. Finally, all the samples were performed with flow cytometry in within 1 hour.

Cell cycle studies

A2780DDP cells were seeded in six-well plates at a density of 1×10^5 per well and incubated at 37 °C for 12 h. cisplatin, doxorubicin, cisplatin+ doxorubicin, NPs were added in the wells at the final concentration of 10 μM of Pt and 5 μM of doxorubicin. After treatment for 24 h, the media was removed and the cells were washed 3 times

with cold PBS. After fixed with 50% ethanol at 4 °C for 12 h, the cells were harvested and treated with RNAse (100 μg /ml) and propidium iodide (100 μg /ml) at 4 °C for 30 min. Finally, all the samples were performed with flow cytometry in 1 h.

The combination index

The Combination Index (CI) was calculated from the equation

$$\mathrm{CI} = \frac{CA,x}{ICx,A} + \frac{CB,x}{ICx,B}$$

CA, $_X$ and CB, $_X$ represented the concentrations of drug A and drug B used in combination to reach x% drug effect respectively. IC_X,A and IC_X,B represented the concentrations for single drug to reach the same effect. A CI of less than, equal to, and more than 1 suggested synergy, additivity, and antagonism, respectively.

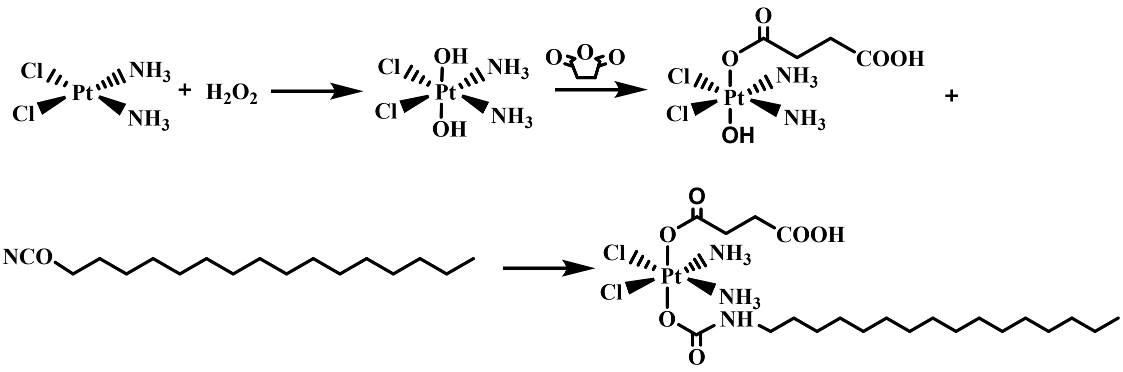

Scheme S1. The synthesis of C_{16}-Pt (IV)-COOH.

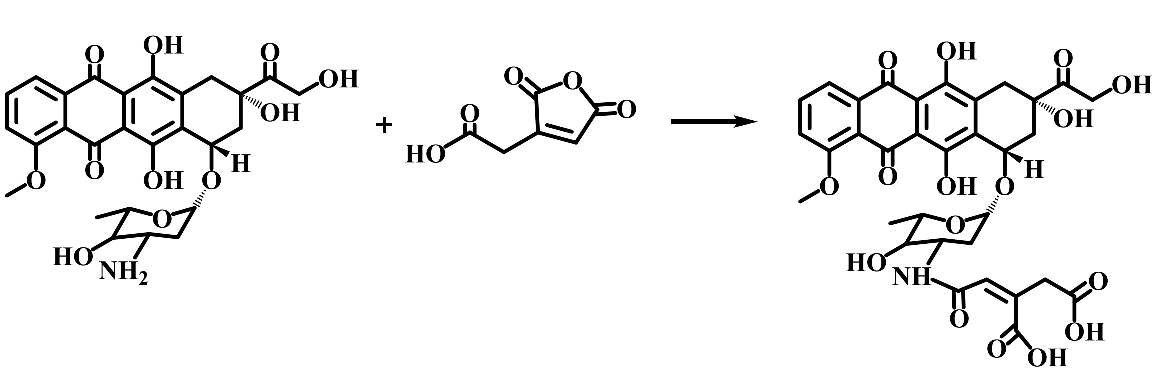

Scheme S2. The synthesis of CAD.

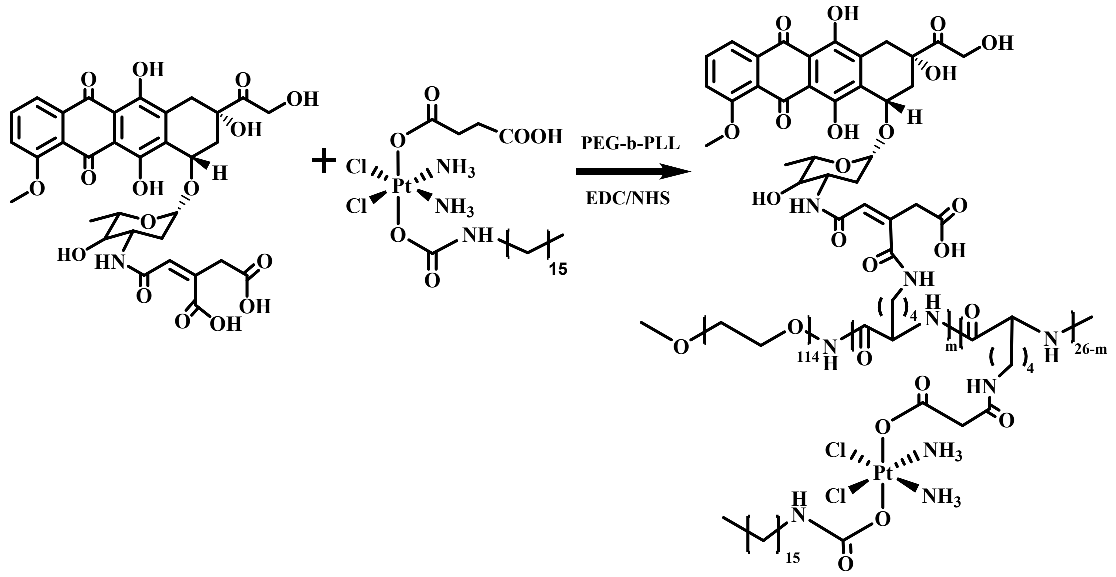

Scheme S3. The synthesis of PEG_{114}-b-PLL_{26}-Pt (IV)-CAD.

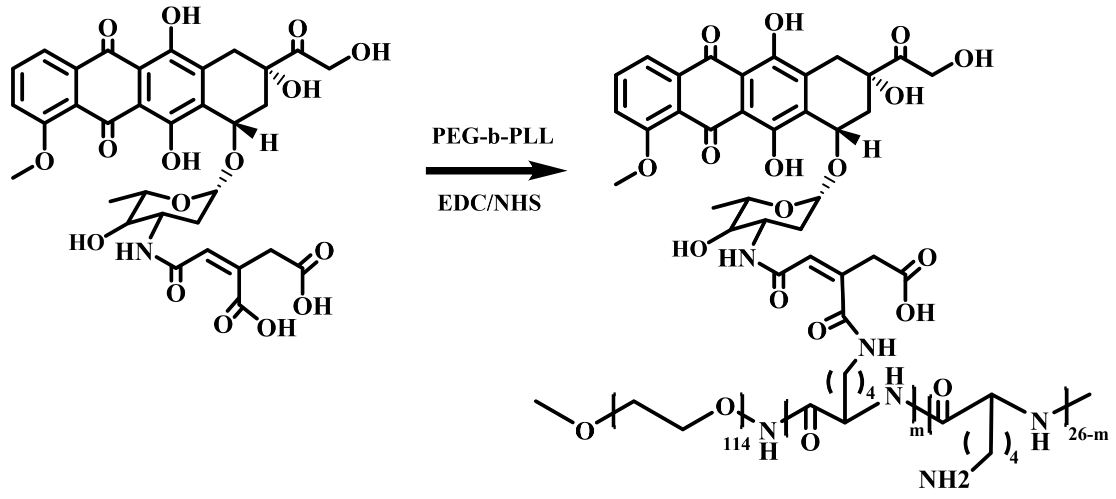

Scheme S4. The synthesis of PEG_{114}-b-PLL_{26}-CAD.

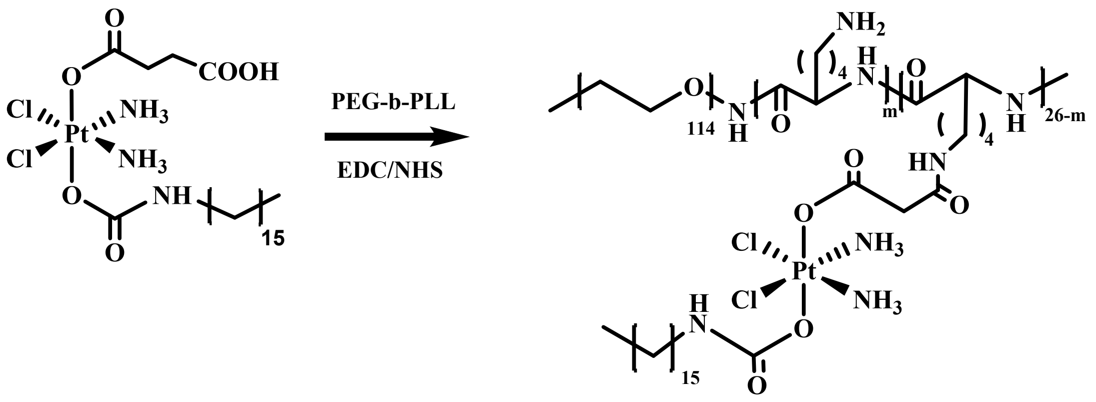

Scheme S5. The synthesis of PEG_{114}-b-PLL_{26}-Pt (IV).

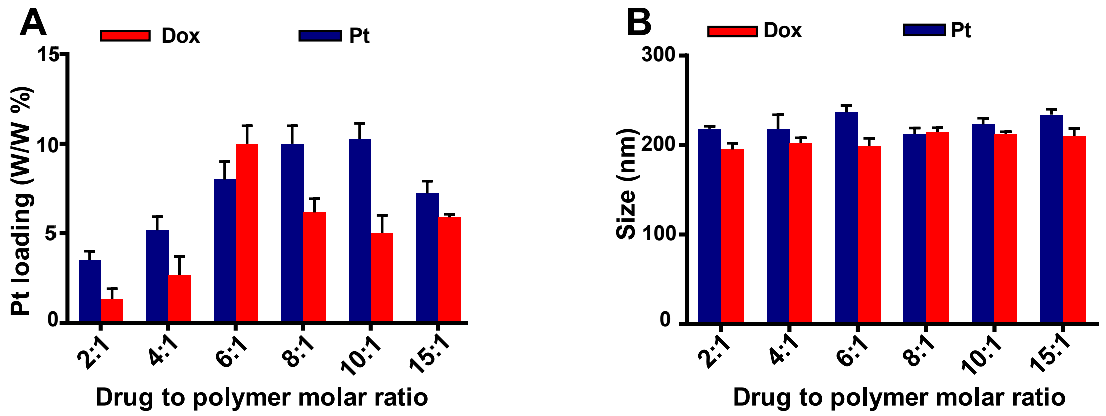

Figure S1. Formulation optimization of the nanoparticles. Pt and Dox loading (A), size (B) were shown according to Pt and Dox to molar ratios.

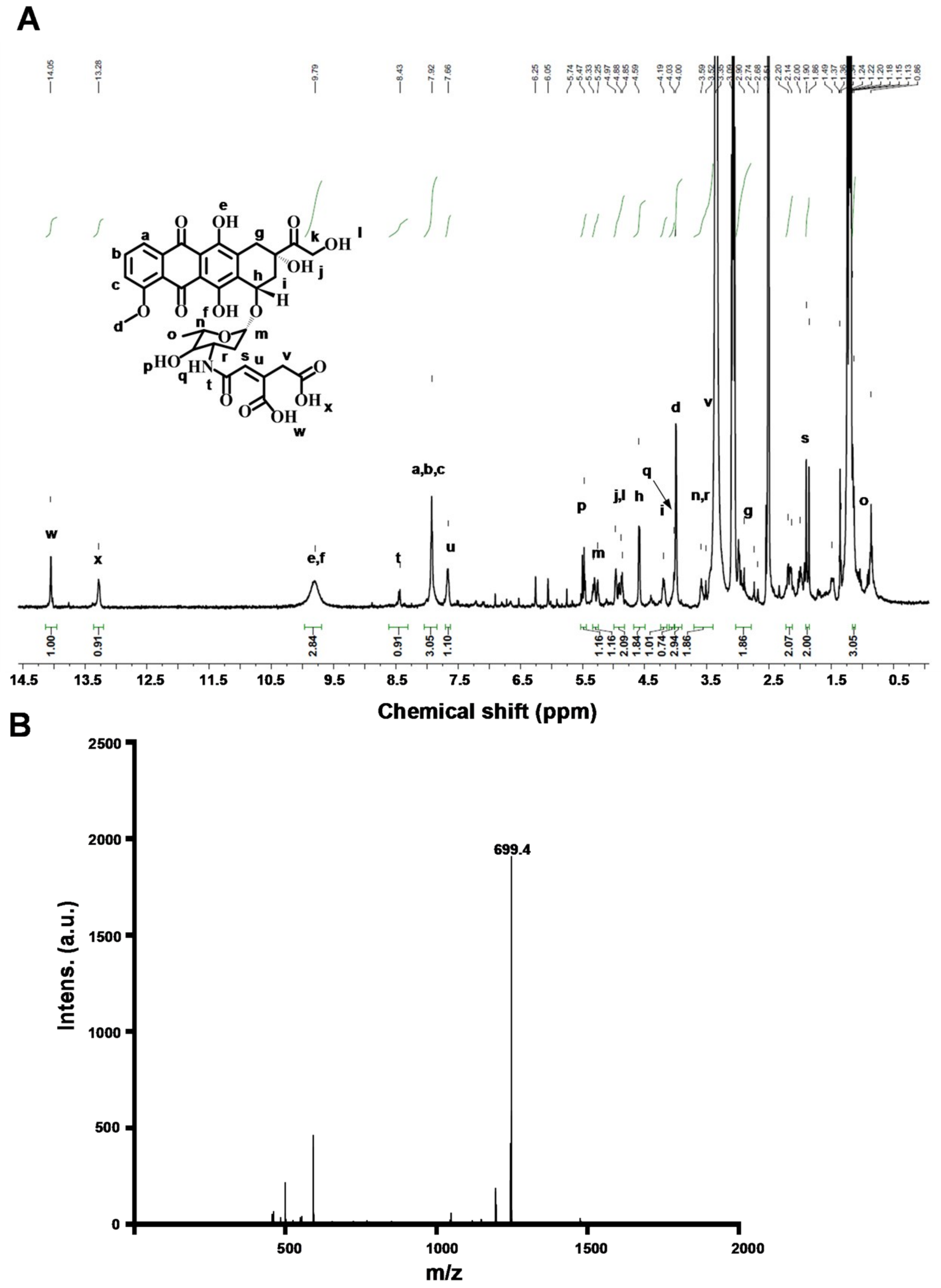

Figure S2 ^{1}H NMR spectrum of CAD in DMSO-d_6(A), and characterization of CAD by MALDI-TOF (B).

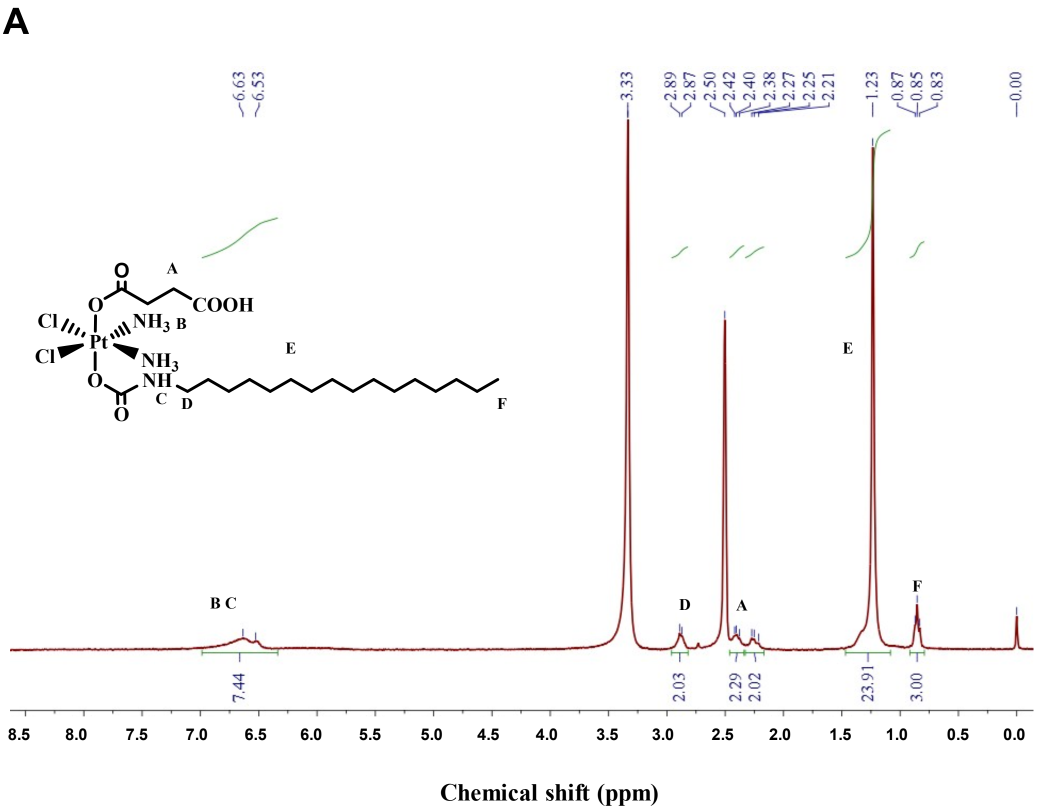

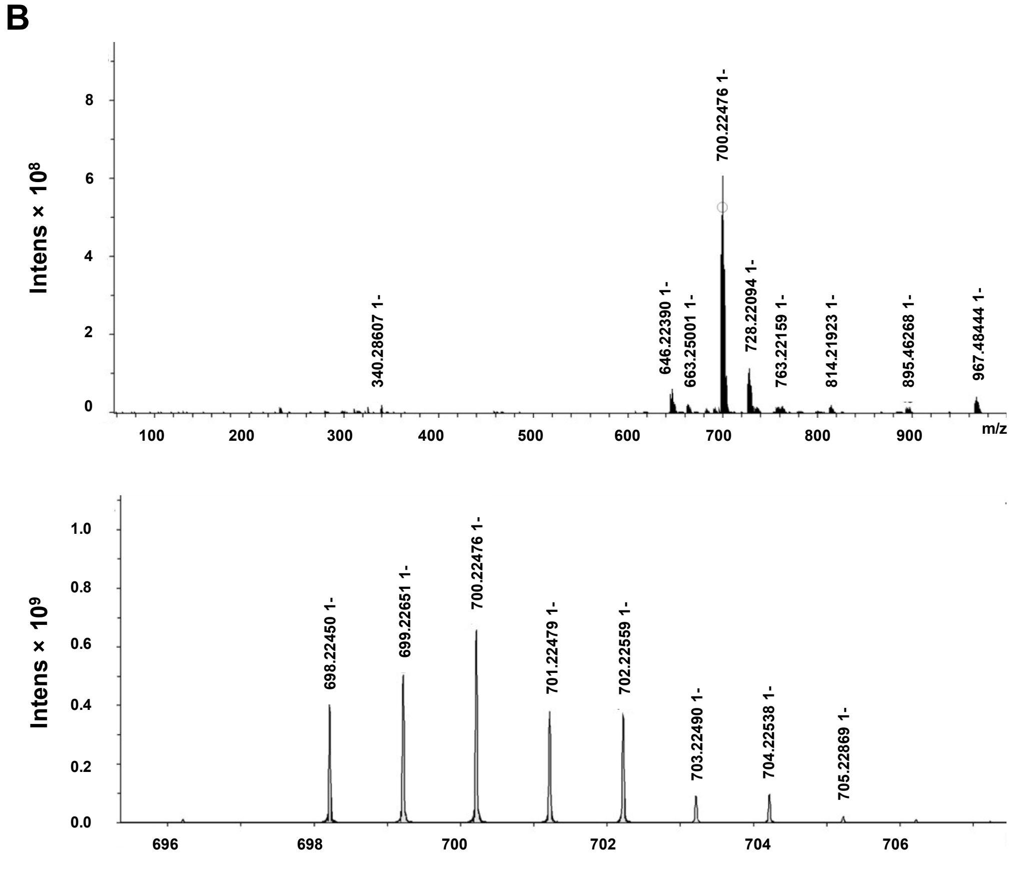

Figre S3. ^{1}H NMR spectrum of Pt (IV) in DMSO-d_6(A), and characterization of

Pt (IV) by HRMS (ESI) (B).

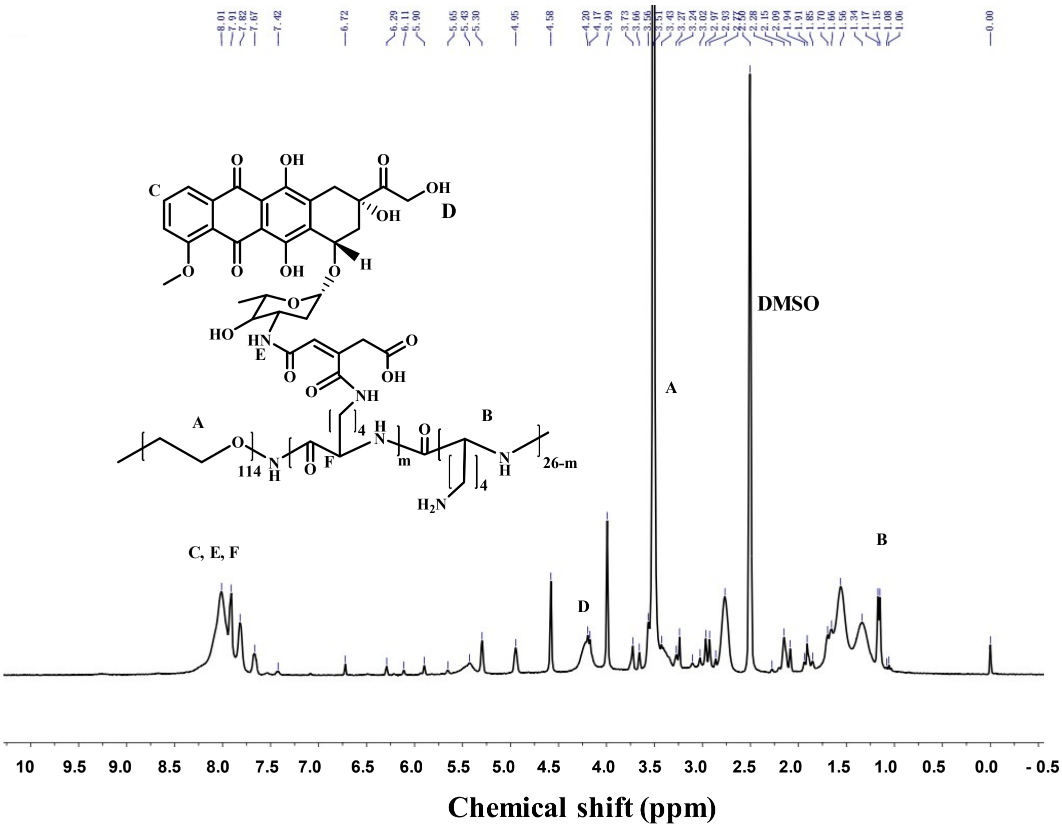

Figure S4. ¹H NMR spectrum of PEG$_{114}$-b-PLL$_{26}$-CAD.

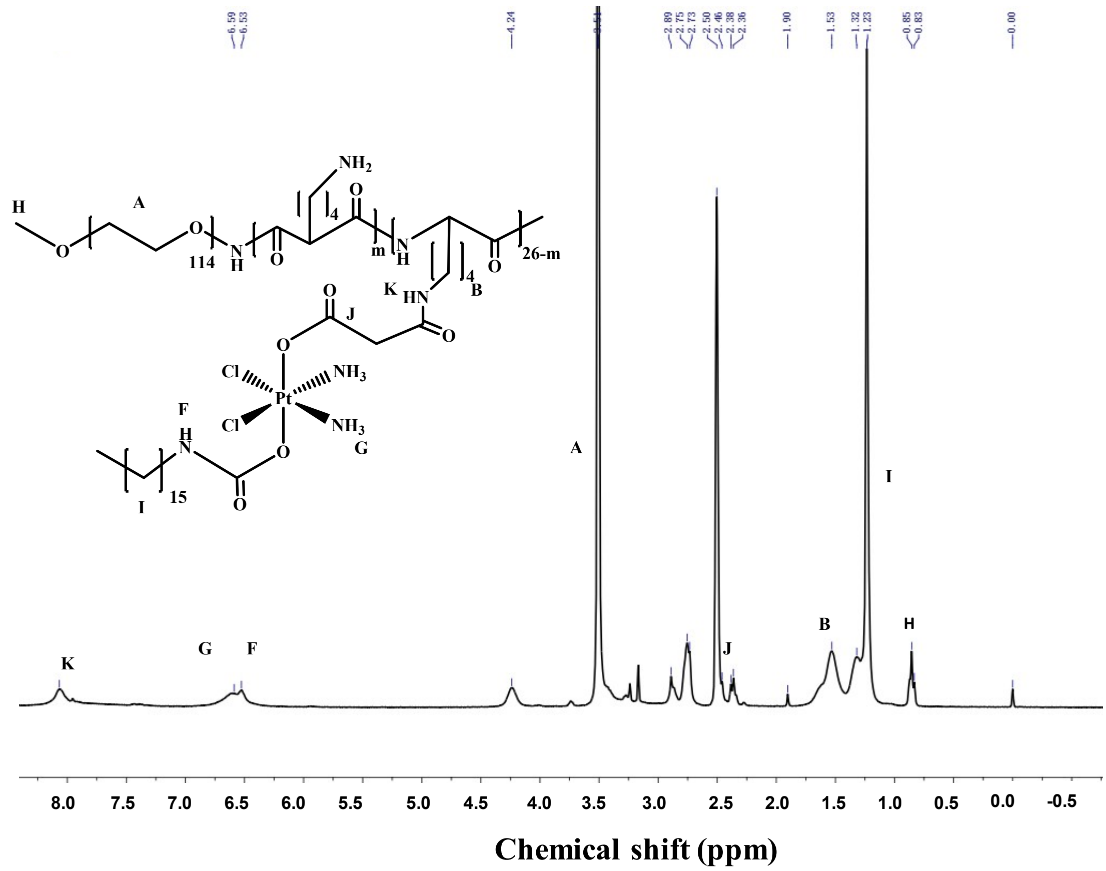

Figure S5. ^{1}H NMR spectrum of PEG_{114}-b-PLL_{26}-Pt (IV).

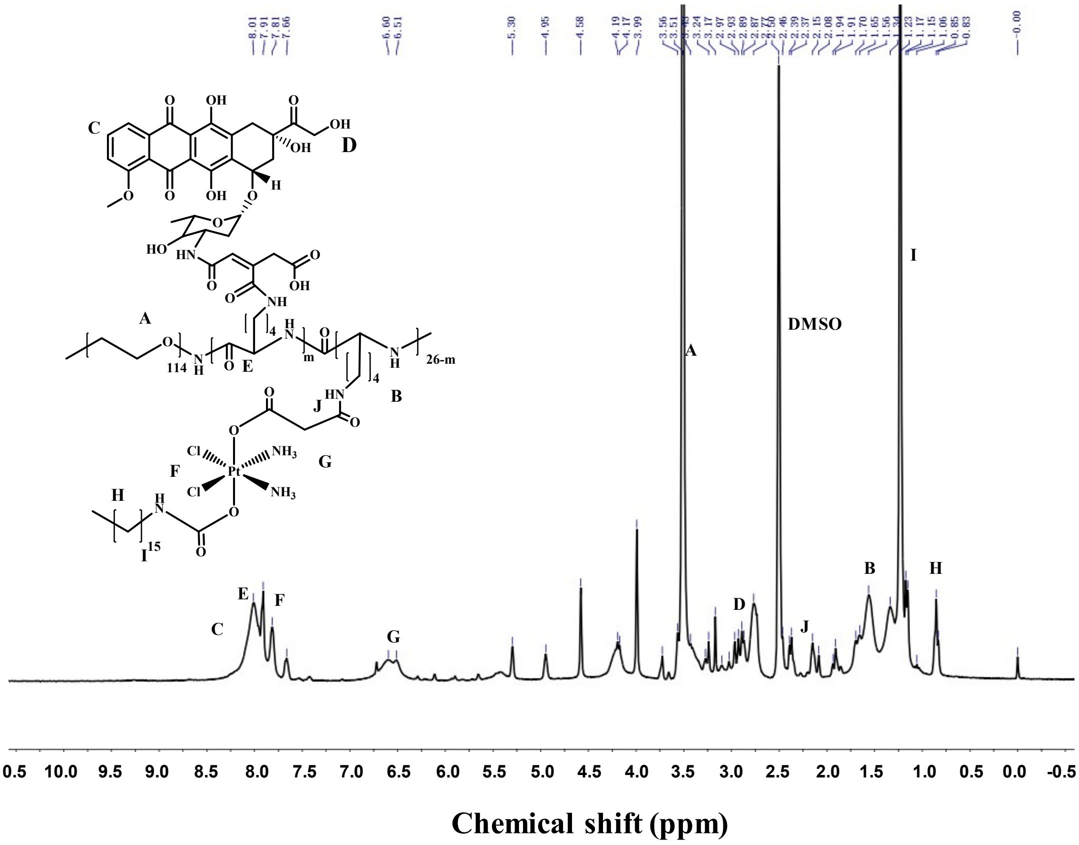

Figure S6. ^{1}H NMR spectrum of PEG$_{114}$-b-PLL$_{26}$-Pt (IV)-CAD.

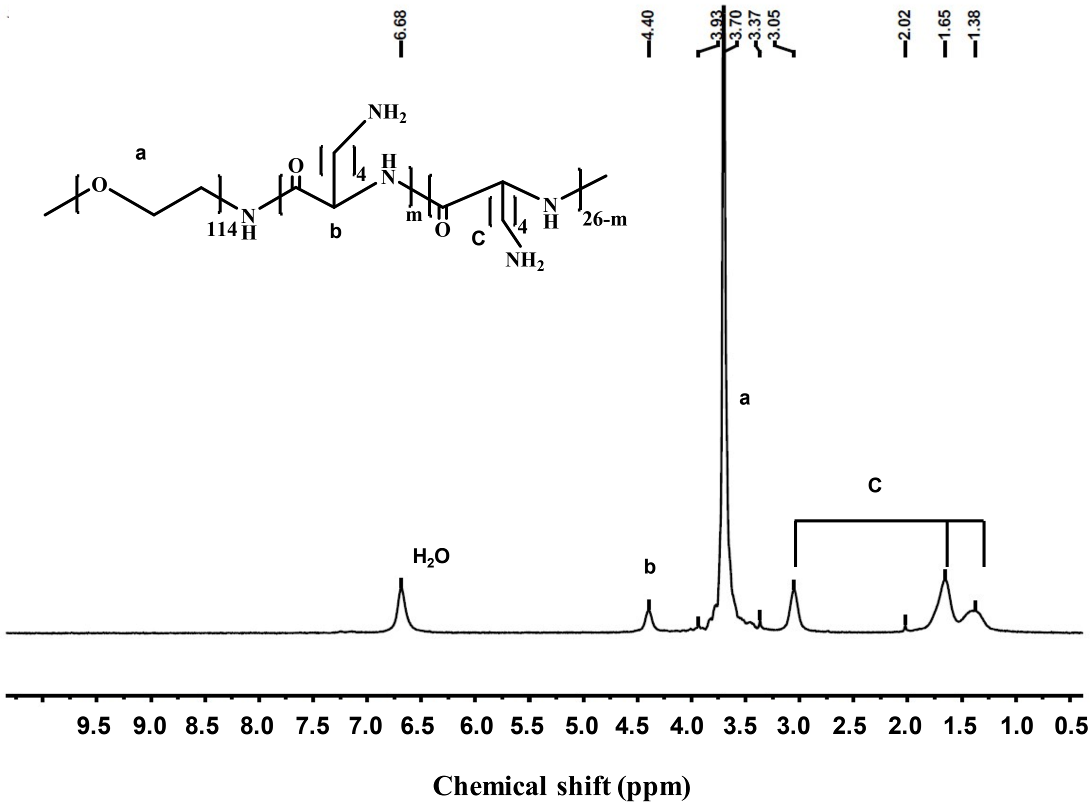

Figure S7. ^1H **NMR spectrum of** PEG_{114}**-b-**PLL_{26}**.**

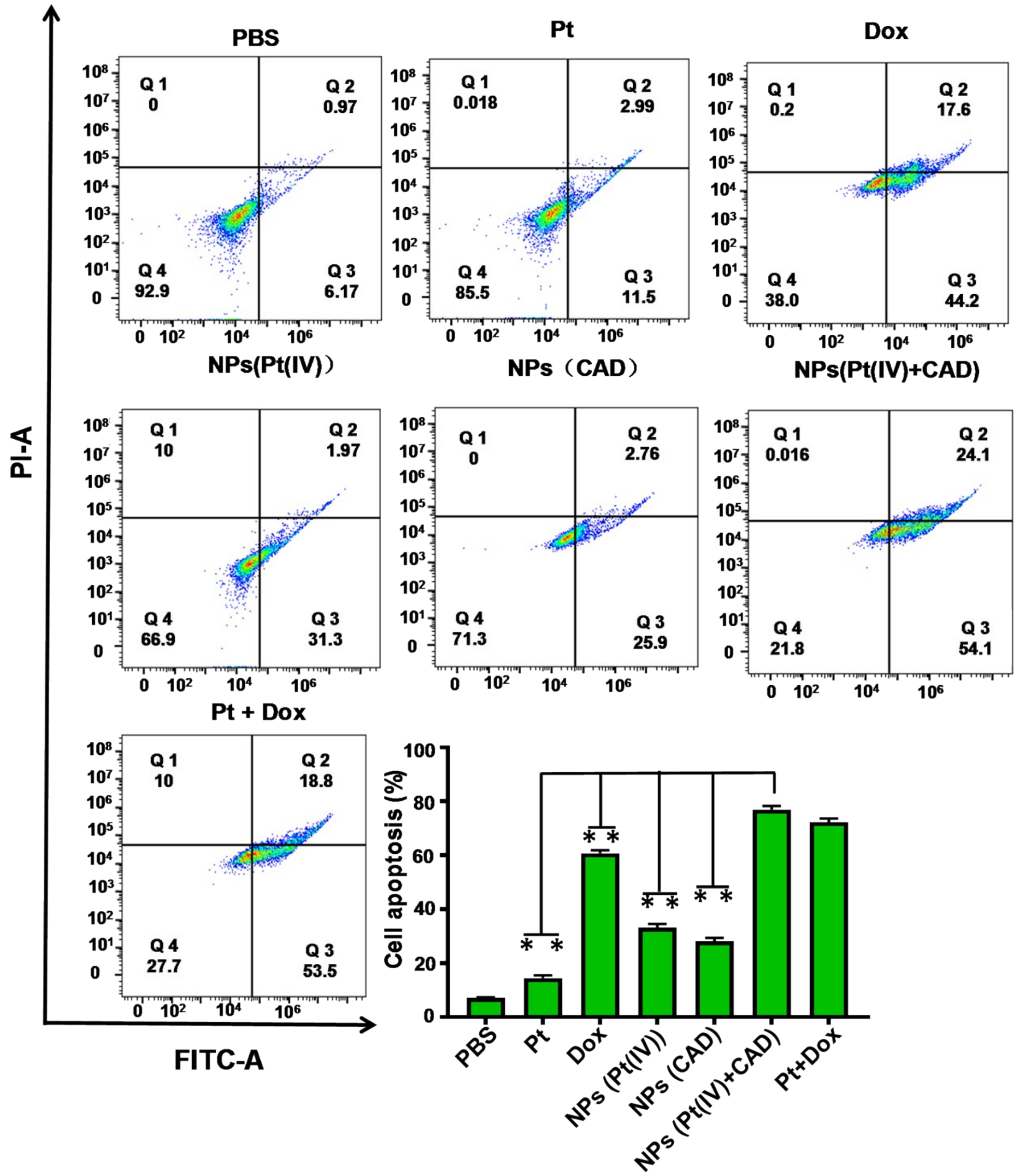

Figre S8. Cell apoptosis of Pt, Dox, and nanoparticles in A2780. The cell apoptosis induced by Pt + Dox, NPs (Pt (IV)+CAD), NPs (Pt (IV)), Pt, NPs (CAD), Dox and, PBS**.** Significance is defined as **P<0.001, *P<0.01.

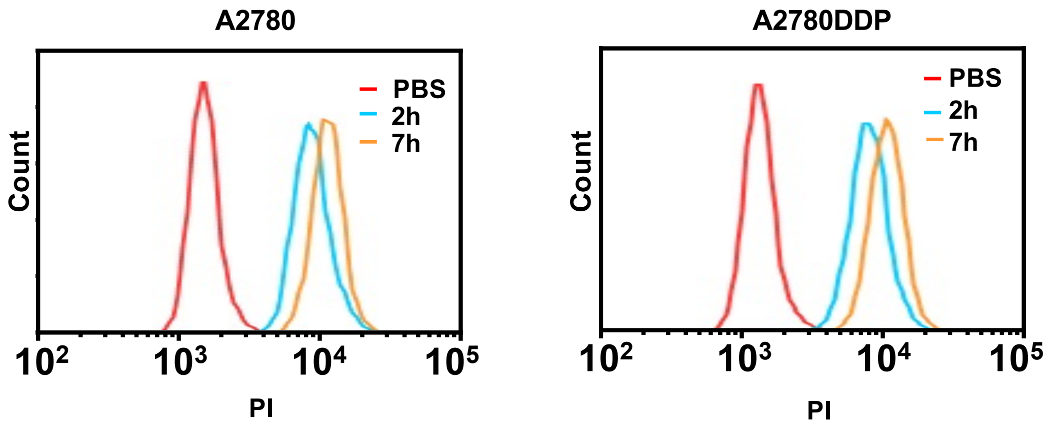

Figure S9. The Dox uptake in A2780 and A2780DDP. To quantify the NPs (Pt (IV)+CAD) uptake, flow cytometry was used to monitor the fluorescence intensity in the A2780 and A2780DDP cells at 2 h and 7 h.

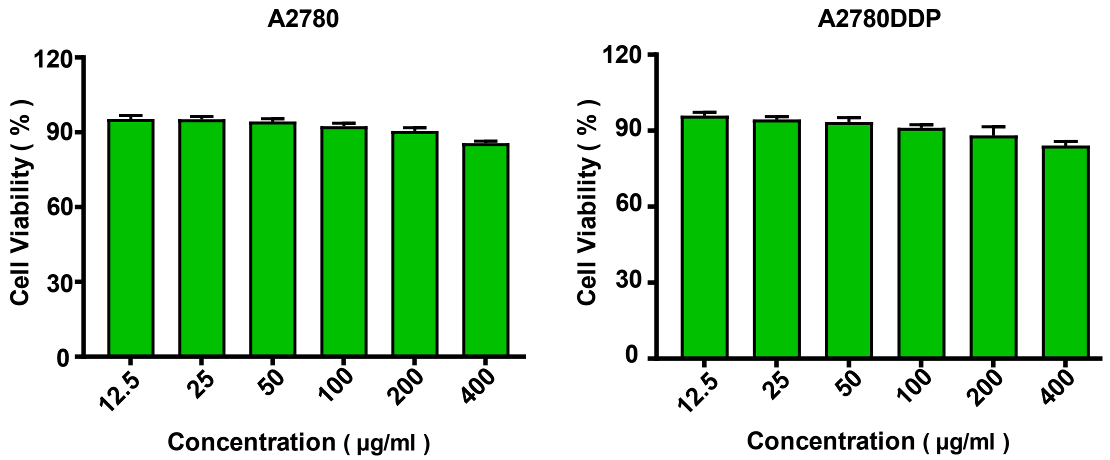

Figure S10. The dose-responsive cytotoxicity of PEG_{114}-b-PLL_{26} at 48 h
